# Understanding the Molecular Circuitry of Cell Lineage Specification in the Early Mouse Embryo

**DOI:** 10.3390/genes2030420

**Published:** 2011-07-13

**Authors:** Anna Bergsmedh, Mary E. Donohoe, Rebecca-Ayme Hughes, Anna-Katerina Hadjantonakis

**Affiliations:** 1 Center for Reproductive Medicine and Infertility, Weill Cornell Medical College, New York, NY 10065, USA; E-Mail: anna.bergsmedh@me.com (A.B.); 2 Burke Medical Research Institute, White Plains, NY 10605, USA; E-Mail: reh2010@med.cornell.edu; 3 Departments of Neuroscience and Cell and Developmental Biology, Weill Cornell Medical College, New York, NY 10065, USA; 4 Developmental Biology Program, Sloan-Kettering Institute, New York, NY 10065, USA

**Keywords:** mouse embryo, early development, lineage specification, epigenetic marks, transcriptional circuitry, stem cells

## Abstract

Pluripotent stem cells hold great promise for cell-based therapies in regenerative medicine. However, critical to understanding and exploiting mechanisms of cell lineage specification, epigenetic reprogramming, and the optimal environment for maintaining and differentiating pluripotent stem cells is a fundamental knowledge of how these events occur in normal embryogenesis. The early mouse embryo has provided an excellent model to interrogate events crucial in cell lineage commitment and plasticity, as well as for embryo-derived lineage-specific stem cells and induced pluripotent stem (iPS) cells. Here we provide an overview of cell lineage specification in the early (preimplantation) mouse embryo focusing on the transcriptional circuitry and epigenetic marks necessary for successive differentiation events leading to the formation of the blastocyst.

## 1. Why Study the Mouse Embryo?

The procurement and delivery of properly fated replacement cells is an exciting area of current biomedical research and offers great hope for patients with degenerative and other diseases. Embryonic stem (ES) cells and induced pluripotent stem (iPS) cells possess the ability to differentiate into all the cell types of an adult. This differentiation potential into all lineages of the embryo-proper and fully developed organism is referred to as pluripotency. However, pluripotent ES cells and iPS on their own cannot give rise to the entire conceptus, as they lack the potential to develop many of the extraembryonic tissues that support the development and patterning of the embryo-proper. Thus, all three cell lineages of the blastocyst are required both for embryo development and survival.

Studies in cellular replacement therapies have made tremendous advancements due to seminal studies using the preimplantation mouse embryo as a model organism. Work in the mouse also provides a biological tool to generate and correct genetic mutations. In many instances gene mutations in the mouse produce similar phenotypes to human disease mutations thereby providing insight into the molecular mechanisms regulating both embryonic development and disease progression and drawing parallels between these two seemingly disparate processes. Furthermore, mouse genetics has allowed epistatic pathways to be defined.

Without the pioneering studies in mouse, regenerative medicine may not have made the tremendous progress of obtaining somatic cells and reprogramming them back to the early pluripotent state for the future repopulation of damaged tissues. However, many questions remain unanswered. How does the embryo control cell lineage choice? How can differentiated adult somatic cells acquire an iPS state? Orchestrations of transcriptional and epigenetic circuits define the state and thus can control the destiny of cells into the proper cellular fate. Here, we review some of the observations that define early events of cell lineage determination in the early mouse embryo.

## 2. Preimplantation Is Devoted to the Generation and Expansion of Extraembryonic Tissues

Mammalian development can be divided into pre- and a postimplantation phases, where the first serves to prepare the embryo for attachment to the uterine wall for further growth and development. Within the first four or so days of development, the lineages destined to form the embryo-proper as well as the extraembryonic tissues, essential for *in utero* survival, are specified and spatially segregated [1,2]. Preimplantation development is a phase that is unique to placental mammals and involves two sequential cell fate decisions giving rise to the three distinct lineages; the pluripotent epiblast (EPI), as well as two extraembryonic tissues, the trophectoderm (TE) and the primitive endoderm (PrE).

**Figure 1:**
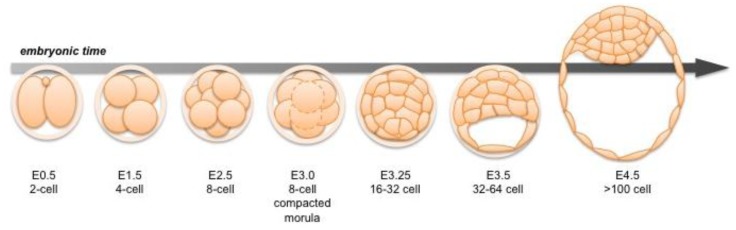
Mouse preimplantation development. A schematic representation and overview of mouse preimplantation development during embryonic day (E) 0.5–4.5. Following fertilization the early mouse embryo undergoes a series of cleavages to generate the lineages necessary for *in utero* survival.

Following fertilization, the zygotic genome of the mouse embryo is activated at around the 2-cell stage, a time when maternal mRNAs are also being expressed [3,4]. Transformation of the 2-cell embryo to the 16-cell compacted morula (generally corresponding to embryonic day (E) 3.0) involves a series of ordered cleavage steps (Figure 1). As the embryo develops and its constituent cell numbers increase, developmental potential of individual blastomeres decreases. Individual blastomeres are generally considered totipotent only at the 2-cell stage [5,6], though pluripotency is retained up until the 16-cell stage [7]. Notably, it has been shown that blastomeres of 4-cell stage embryos differ in their individual developmental potential, according to their spatial distribution and cleavage patterns [8,9]. Individual blastomeres of an 8-cell embryo have the ability to contribute to all three blastocyst cell lineages in chimeras, but they are generally unable alone to support full-term development of embryonic and extraembryonic tissues [10–13].

**Figure 2:**
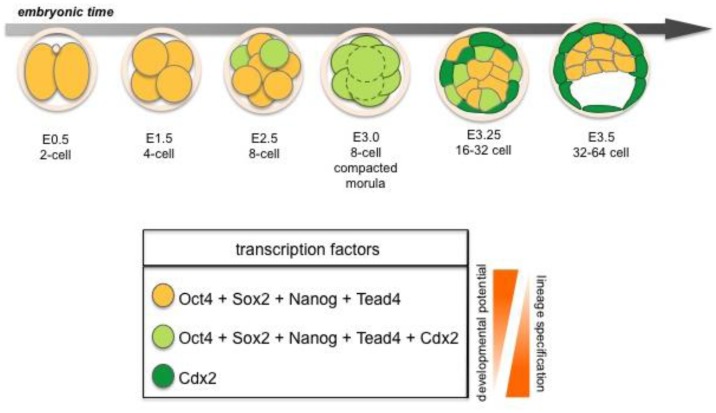
The TE *vs.* ICM cell fate decision in the mouse embryo. This cell fate decision takes place during the transition from morula (E2.5) to early blastocyst (E3.5). Here regulation of lineage specific transcription factors such as Cdx2, Tead4, Nanog, Oct4 and Sox2, will result in segregation of the first cell lineage trophectoderm (TE) from inner cell mass (ICM).

The TE is the first cell lineage to emerge and will give rise to the embryonic portion of the placenta. The TE is positioned on the surface of the embryo where it forms a specialized epithelium. The initial differentiation of the TE integrates morphogenesis with lineage specification as TE specification occurs concomitant with epithelialization likely induced by cell polarization as well as up-regulation of lineage-specific transcription factors such as Cdx2 during the early 8-cell morula stage [14,15] (Figure 2 and 7). At the 32-64-cell blastocyst stage the embryo forms a cavity, the blastocoel, and at this point it comprises an outer TE cell layer and an inner cell mass (ICM).

**Figure 3:**
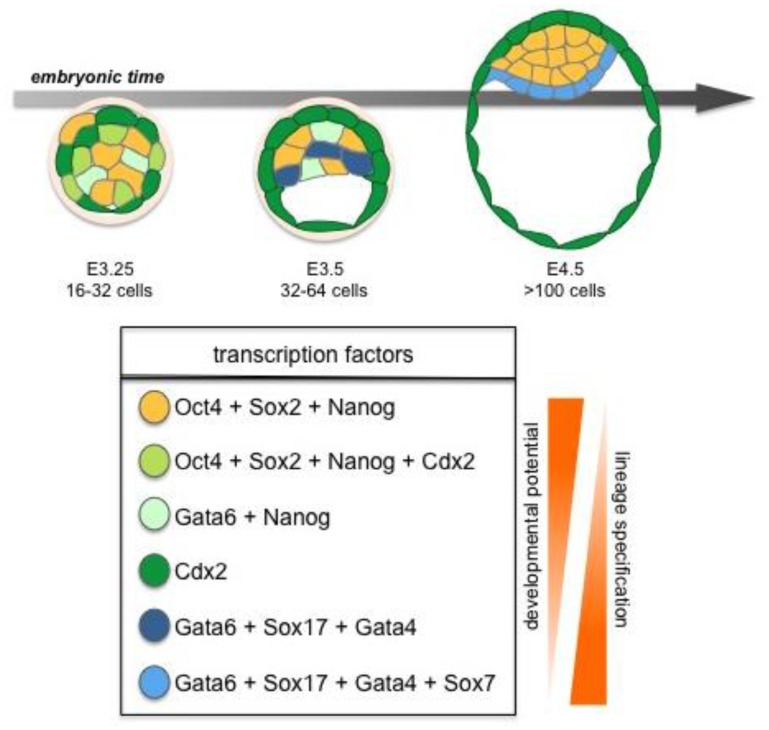
Cell commitment to pluripotent epiblast (EPI) *vs.* primitive endoerm (PrE) coincides with sequential expression key *trans*-factors. This second cell fate decision involves three successive phases. From the initial co-expression, emerges a mutually-exclusive expression of *trans*-factors and salt-and-pepper distribution of cells fated to form (Nanog-positive) EPI or (Gata6/Sox17/Gata4-positive) PrE (∼64-cell stage), followed by cell sorting (>100 cell stage) which achieves a positional segregation of EPI *vs.* PrE fated cells into different tissue layers within the ICM. At the time of implantation the Gata6/Sox17/Gata4/Sox7-positive PrE cell layer lies adjacent to the blastocoelic cavity, while the EPI is internal being encapsulated by PrE at one side and TE at the other.

Later, the ICM will give rise to two cell lineages, the pluripotent EPI, and the PrE, which will give rise to the endodermal component of the visceral and parietal yolk sacs (Figure 3) [2]. Together the three lineages of the late blastocyst: TE, PrE and EPI will produce both the embryo-proper (EPI) as well as its ancillary extraembryonic tissues (TE and PrE), which serve as a maternal-fetal interface as well as guiding the development of a functional and viable embryo [2,16,17].

## 3. Each Lineage in the Pre-Implantation Embryo has Its Own Progenitor Cells

The three different cell lineages of the blastocyst can each be harnessed *in vitro* through the derivation of lineage-specific self-renewing stem cells from blastocyst stage embryos. Trophoblast Stem (TS) cells are derived from and represent TE lineage, and eXtraembryonic ENdoderm (XEN) cells represent the PrE [18,19]. Pluripotent Embryonic Stem (ES) cells derived from and representing the EPI are the best studied stem cells in both mouse and man [20-22]. These three cell types can be propagated *in vitro* under conditions of “stemness” and also directed to differentiate, thereby providing additional tools for studying the gene regulatory and signaling networks operating and co-operating within the lineages of the early mouse embryo.

## 4. How Are Genes Controlled at the Transcriptional Level?

Lineage-specific *trans*-factors binding to their cognate DNA binding motifs are critical in the regulation of cell fate in the early embryo. Thus, the mechanism by which these *trans*-factors operate and the regulatory regions they target in lineage allocation is of great interest. Here, we provide a basic overview of how genes are regulated and then review what is known about lineage-specific transcription and chromatin packaging in the early embryo.

Genomic DNA provides information in two ways. In addition to specifying the sequences of protein-encoding mRNAs, genomic DNA consists of regulatory regions for transcription factors to bind and control the levels of gene expression [23]. These regulatory regions consist of RNA polymerase II (RNAP II) promoter regions directly upstream of the transcriptional start sites as well as gene-distal enhancers and repressors. Regulatory regions provide cell-specific gene activity and a complexity necessary for the surprisingly low (∼23,000) number of protein-coding genes in man and mouse. Because a single metazoan genome specifies many distinct cell types in the adult, there is an ordered process of activating and repressing the appropriate genes during development. Mouse transgenics coupled with cell culture experiments have identified these distant genic regulatory regions known as enhancers [23,24]. Promoter and enhancer interactions may be close range or very long range as in the *β-globin* and *sonic hedgehog (shh)* gene [23,25,26]. Gene expression occurs by *trans*-factors capable of DNA bending and looping to position distal enhancers to the promoter of a gene in a particular chromosomal territory. This long-range regulatory region communication is facilitated by chromatin insulators, DNA bending trans-factors and/or matrix attachment proteins [27]. Dependent on whether the gene is to be activated or repressed is reflective of the transcription factors bound and the open or closed chromatin configuration [23]. The early mouse embryo as well as embryo-derived stem cells, provide favorable systems to study the regulatory networks necessary for the dynamics of cell lineage specification.

## 5. Chromatin States in the Developing Embryo

**Figure 4:**
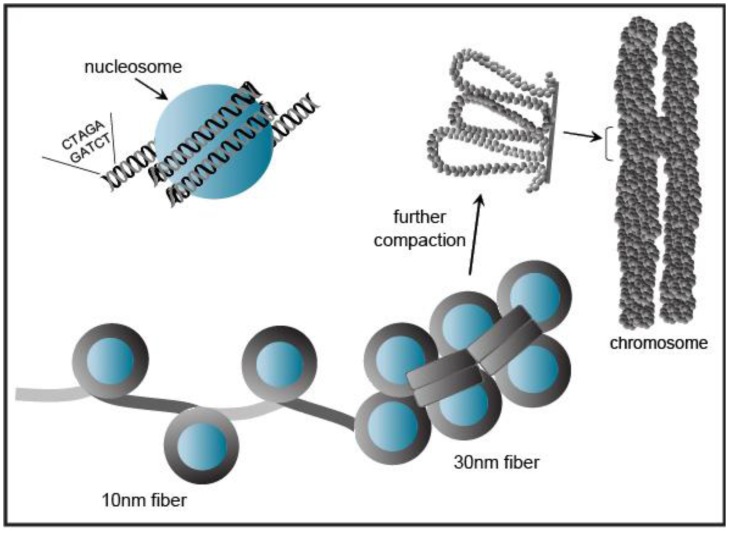
DNA is packaged into chromatin. About 80% of genomic DNA is packaged into nucleosomes, which consists of 146 base pairs of DNA wrapped around a histone octamer. The chromatin fibers adopt a “beads on a string” configuration with a diameter of 10 nm and may be further condensed into a compact 30 nm fiber. The chromatin is folded into a defined structure (as defined by the basic radial loop model) by chromatin remodeling enzymes during cell mitosis.

Eukaryotic genomic DNA does not exist in a linear manner, but remarkably the several meters of DNA in a given cell is compacted to fit into the volume of a single nucleus [28]. This tremendous packaging of DNA into chromatin must still allow access for DNA-binding factors to regulate transcription, as well as the DNA-dependent processes of replication, repair, and recombination. Chromatin wraps DNA, RNA, histone, and non-histone proteins into a complex known as a nucleosome (Figure 4). The nucleosomal complex consists of 146 base pairs of DNA wrapped around a core histone octamer (two copies each of histones H2A, H2B, H3, and H4) into a higher-ordered chromatin structure. Nucleosomal arrays *in vitro* adopt a “beads on a string” fiber with a diameter of 10 nm that may be condensed to a 30 nm fiber [28] (Figure 4).

**Figure 5:**
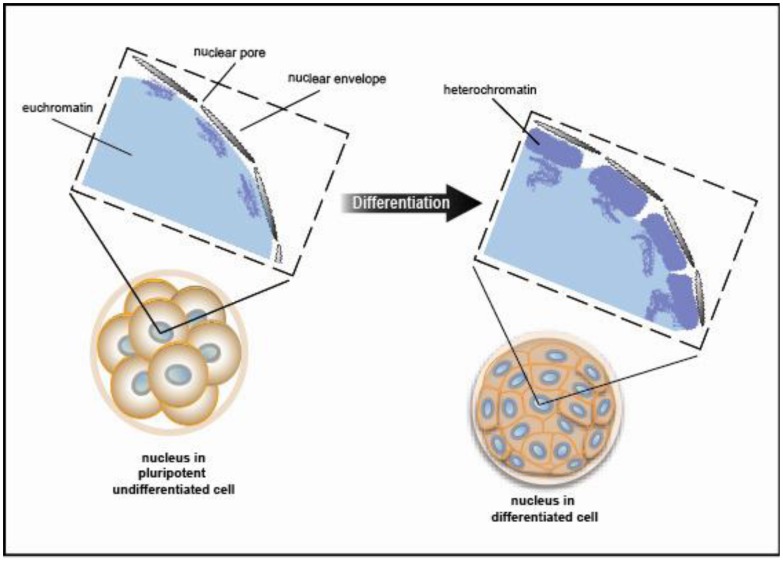
Changes in chromatin fiber density may distinguish pluripotent and differentiated cell types. Schematic view (interpreted from the experimental data in reference [29]) of nuclei from pluripotent cells (left) and differentiated cells (right) using electron spectroscopic imaging to visualize the chromatin organization. Undifferentiated cells have a less compact, more euchromatin (open chromatin structure) as compared with the heterochromatin (closed chromatin structure) present in differentiated cells.

Chromatin exists in a condensed or inactive state known as heterochromatin and in a decondensed or open state known as euchromatin. A recent study using electron spectroscopic imaging to visualize chromatin organization in both the early pre-implantation mouse embryo and undifferentiated cell types, including iPS cells, revealed that chromatin fibers comprise an open configuration of 10 nm mesh that fill the nucleus perhaps reflecting genes poised and ready for activation [29,30]. Upon cellular differentiation, the chromatin structure becomes more condensed leading to large regions of the nuclear volume devoid of DNA. (Figure 5 shows a schematic view of the data presented in [29]). This open *vs.* closed chromatin state reflects chromatin modifiers and *trans*-factors that regulate fiber compaction. Taken together, changes in chromatin fiber density may serve as a mark for pluripotency.

**Figure 6:**
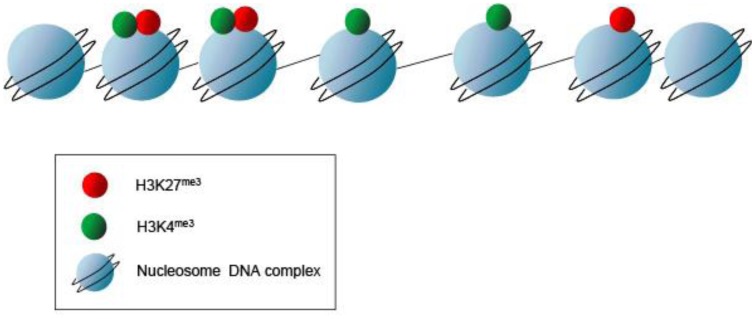
Post translation modifications (PTMs) occur on the histone tails and a PTM code designates genes “on” or “off”. In early embryos and ES cells, modifications can dictate gene activation or repression. One example is the bivalent marks found in undifferentiated ES cells in which histone H3 lysine 27 is trimethylated (H3K27me3) and histone H3 lysine 4 is trimethylated (H3K4me3). H3K27me3 is typically a repressive mark whereas H3K4me3 denotes an active gene.

Not only does the mechanical chromatin packaging determine heterochromatic *vs.* euchromatic state but covalent post-translational modifications (PTMs) on the nucleosomal histone tails may identify active genomic regions (Figure 6). Histone PTMs may provide a fundamental way of regulating DNA accessibility during gene transcription, DNA replication, and DNA replication, and DNA damage repair [31] Methylation of histones may occur at multiple lysine and arginine residues. Up to three methyl groups at each lysine may be produced. For example, histone H3 lysine 4 trimethylation (H3K4me3) typically denotes a transcriptionally active genomic region, whereas histone H3 lysine 27 trimethylation (H3K27me3) signifies an inactive or repressed region. These repressed regions in early development are genes involved in pluripotency and lineage differentiation [32–34]. These PTMs provide a “landing pad” or scaffold for transcriptional co-activators or co-repressors. But, this combinatorial pattern of histone marking is rather complex. Although ES cells show a globally open chromatin structure and are enriched for active H3K4 methylation marks, only a subset of promoters with H3K4 methylation show enrichment for elongating RNAP II and histone H3 lysine 36 di- or tri-methylation (H3K36 me2 or me3) signifying active transcription through the loci. Histone PTMs are some of the earliest marks of linage segregation with both the ICM and TE displaying differential histone modifications [35]. In the ICM, the *Oct4* promoter has increased histone 4 lysine 8 acetylation (H4K8ac) and H3K4me3 [36]. This contrasts to the *Oct4* promoter in the TE, which has increased H3K9me2 (a repressive mark). Torres-Padilla, et al describe other marks, histone 3 arginine 17 and arginine 26 monomethylation (H3R17me and H3R26me), that differ in the mouse blastomeres as early as the 4-cell stage [37]. They show that there are higher levels of H3R17me and H3R26me in cells destined to become the ICM, whereas, lower H3R17me and H3R26me in the TE fated cells. Ectopic expression of Carm1 (the H3-specific arginine methyltransferase responsible for placing the H3R17 and H3R26 methylation marks) into one of the blastomeres at the 2-cell embryo stage shows that both the *Nanog* and *Sox2* pluripotent genes get up regulated and fated to ICM [37]. Thus, PTMs such as histone arginine methylation may mark the cellular fate decisions towards pluripotency in the early mouse embryo. Additional PTMs as well as the writers and readers of these marks may also signify differential cell fates.

Studies in both mouse and human ES cells using chromatin immunoprecipitation (ChIP) assays show that regions exist with overlapping active and repressive histone PTMs present [38]. These “bivalent marks” (H3K4me3 and H3K27me3) provide signatures for genes poised and ready for activation, and reflect the relatively euchromatin signature of the undifferentiated state (Figure 6). Interestingly, a highly significant number of these bivalent domains co-localize with binding sites for the pluripotent factors Nanog and Oct4 [33]. Thus, the bivalent chromatin state is suggested to be a mechanism for retaining chromatin and cellular plasticity in early development [33].

Not only are there proteins responsible for adding the histone modifications, but there are factors that interpret or read the marks, and other proteins that remove the covalent marks, thus providing a dynamic process [31]. A recent report shows that LSD1 (lysine-specific demethylase 1), a key modifier of H3K4 methylation, co-occupies bivalent domains and regulates the balance between pluripotency and differentiation. LSD1 participates in gene repression by enzymatically demethylating H3K4 mono- and di-methyl (me1/me2) marks. Knockdown of *LSD1* results in differentiation of human embryonic stem cells derepressing several developmental genes [39]. These results suggest that a critical balance is necessary between the pluripotent *trans*-factors and the histone modifications for self-renewal and differentiation. Taken together, demethylases may identify either the chromatin signature or enhancers in genes poised for activation in the undifferentiated state.

Enhancers are key regulatory regions crucial for sequence diversity and cell type specificity. Their importance is underscored in early mouse development and lineage commitment, as they are the cognate binding sites for the lineage-specific transcription factors [23]. Enhancers are situated in decondensed chromatin regions as determined by hypersensitivity to digestion by the nuclease DNase I. DNase I hypersensitive sites (HSs) identify nucleosomes that are excluded or repositioned due to binding of transcription factors, and thereby provide a roadmap for potential regulatory regions. Indeed it is speculated that the human and mouse genomes might each harbor up to a million enhancers [23]. Nuclear architecture also plays a role in gene expression designating RNAP II transcription factories within the subnucleus [23]. Together cell-specific *trans*-factors and chromatin modifiers provide the precise and orderly differentiation of the early mouse embryo lineages.

## 6. Specification of the Embryo Lineages in the Mouse: A Transcriptional Circuitry

Several lineage-specific transcription factors have critical roles in the first three lineages of the early embryo. The *trans*-factors Cdx2, Gata3 and Eomes are required in the TE, whereas Gata6, Gata4, Sox17 and Sox7 are required in the PrE, while Oct4, Sox2, and Nanog function in the EPI lineage [2] (Figures 2 and 3). These factors play key roles in development and mutations in many of the genes encoding them will lead to failure in specification and/or maintenance of the lineages in which they are expressed.

Both Oct4 and Nanog are expressed in all cells of the early embryo from 8- to 32-cell stage. By contrast, levels of Sox2 decrease in the embryo until it reaches its lowest levels at 8-cell stage (morula). At 16-cell stage the level of Sox2 increase again, but at this time expression is restricted to inner cells suggesting Sox2 to be an early marker of inner *vs.* outer cell lineages [40]. However, by the 64-cell stage Nanog (named for the mythological Celtic Tir na nÓg “Land of the ever young”) is exclusively expressed within the ICM in the cells that will form the EPI [25]. Moreover, *Nanog* deficient embryos fail to specify EPI [41,42]. PrE is formed initially in *Nanog* mutant embryos but fails to be maintained, suggesting that crosstalk between EPI and PrE may be required for lineage maintenance *in vivo* [43]. The *trans*-factors Oct4 (containing an octamer DNA-binding domain) and Sox2 (an Sry-box containing protein) have been shown to co-operate in the transcription of downstream targets [44]. Oct4 is crucial for stabilizing pluripotency as deficient embryos fail to generate an inner cell mass (ICM), such that all cells of *Oct4*-deficient embryos adopt a trophoblast fate [45]. Furthermore, a recent study suggests that the kinetics of Oct4 may predict cell lineage patterning in the early mouse embryo as early as the 4-8-cell stage [46]. Thus providing a strong bias for whether morphologically indistinguishable cells will divide asymmetrically or symmetrically. These dynamic behaviors are unrelated to the initial levels of Oct4, and therefore independent of the absolute expression level within each cell [46]. Sox2 is present together with Oct4 in the early blastomeres [47–49], and is later downregulated in cells where primitive endoderm (PrE) formation and epithelialization is initiated. However, unlike Oct4, Sox2 will remain active in TE cells. Disruption of *Sox2* also results in an early lethality supporting a requirement for this factor in the specification of the EPI and extraembryonic ectoderm [50]. Thus, both Oct4 and Sox2 are required to correctly specify the cell lineages of the blastocyst, and loss of either factor produces a failure at implantation.

The Zn-finger protein Gata6 is a marker of extraembryonic endoderm lineages, and has been proposed to be required within primitive endoderm (PrE) [51,52]. Indeed two Gata factors, possibly with redundant function, are critical in specification of an extraembryonic endoderm identity as overexpression of Gata6, or the related protein Gata4, in ES cells, is sufficient to downregulate Oct4 expression and induces a primitive endoderm-like identity [53]. Given the co-expression of Gata6 and Gata4 in embryos, the similar effect resulting from their overexpression in ES cells, and since embryos lacking either Gata factor alone, fail to exhibit profound defects in the primitive endoderm, suggests functional redundancy. Thus double mutants will need to be generated and characterized to reveal the *Gata6/4* null state within the primitive endoderm lineage.

Prior to blastocyst formation the homeodomain transcription factor Cdx2 is initially co-expressed with Oct4 in all blastomeres, though the respective levels of expression of these two factors do not correlate [15,50,54,55]. It was recently noted that molecular differences in the expression levels of Cdx2 can be detected as early as at the 8-cell stage, and that these differences may influence cell fate commitment [56]. Moreover, it has been proposed that Cdx2 influences cell polarity by up-regulating polarity genes such as *aPKC* within individual blastomeres. This leads to an asymmetric distribution of *Cdx2* mRNA and thereby results in asymmetric cell division where daughter cells with low levels of Cdx2 contribute to ICM, whereas cells with high levels of Cdx2 will form TE [56]. Furthermore, high levels of Cdx2 result in downregulation of Oct4 in these outside cells as TE differentiation is initiated [54]. Conversely, in inside cells, Oct4, Sox2 and later Nanog are continuously expressed. The restriction of these transcription factors to inside cells, and of Cdx2 to outside cells, reveals molecular details of what is likely to be the first cell lineage segregation occurring within the developing mouse embryo [54,57].

That said, it was recently reported that Oct4 expression is not restricted to ICM exclusively in early stage bovine blastocysts, but is instead is co-expressed with Cdx2 in the TE. These data therefore suggest an earlier restriction of TE lineage fate in mice compared to cow. These differences in Cdx2 expression and the divergence of early TE lineage restriction between the mouse and cow could be attributed to the fact that mouse embryos implant soon after fertilization. Therefore mouse embryos would require their placenta for sustenance far earlier than other mammals, including the cow as well as humans [58]. Importantly these data reveal that some of the details of the transcriptional circuits regulating early mammal development are divergent between species [59].

## 7. Segregation of TE and ICM Cell Fate: Gene Regulatory Networks Operating in the Early Embryo

Segregation of the two first lineages, TE and ICM, takes place in period of transition from morula to early blastocyst (8 to 16-cell stage). Formation of TE requires: (1) asymmetric cell division, where cell polarization as well as cell position play crucial roles; and (2) tightly regulated gene expression by key *trans*-factors (Figure 2).

The *trans*-factors Cdx2, Gata3 and Eomes have been shown to be important in the TE lineage [15,16,60]. Cdx2 is required for TE lineage maintenance, and is expressed in the outer cells of the blastocyst, where it acts to repress Oct4 and Nanog *trans*-factor levels in these blastomeres. Eomes is also expressed in the TE of the blastocyst, however *Eomes* mutants are reported to arrest at implantation at a later stage than Cdx2 mutants. Cells lacking *Cdx2* or *Eomes* expression do not differentiate towards the TE lineage and continue to express high levels of Oct4 and Nanog respectively, thus these cells maintain characteristics of pluripotency. Overexpression of Cdx2 induces ES cells to differentiate into trophoblast stem (TS) cell like cells [54]. Furthermore, overexpression of Cdx2 in individual blastomeres of early mouse embryos will initiate their differentiation towards TE [56] (Figure 2). Moreover, the transcription factor Gata3 has been shown to be upregulated and specifically expressed within the TE lineage at the blastocyst stage [61]. Although Gata3 is co-expressed with Cdx2 and is capable of inducing ES cells towards a trophoblast state, stable TS cell lines cannot be recovered from ES cells induced to express Gata3. Therefore Gata3 is believed to be necessary for promoting trophoblast maturation, but not for stabilizing TS stem cells [62]. Several studies have shown that the regulation of TE formation is a complex process [3,4,49,56,63] as neither *Cdx2* nor *Eomes* mutants completely fail to initiate TE formation. Consequently, an upstream transcription factor has been suggested to operate in TE lineage formation.

Tead4 (TEA Domain/transcription enhancer factor family) is widely expressed from the 2-cell stage onwards and has been proposed to act upstream of Cdx2 to regulate TE formation [64,65]. *Tead4* mutant embryos exhibit defects in the specification and development of TE and consequently do not form a blastocoel. The expression of Cdx2 is decreased but not depleted in *Tead4* mutant blastomeres [64,65], placing Tead4 transcriptionally upstream of Cdx2. However, recent studies suggest that any Cdx2 detected in *Tead4* mutants could be provided from a maternal pool of *Cdx2* mRNA [3,4] Although, there is no clear indication of the exact function of this maternal pool of *Cdx2* mRNA, it has recently been suggested that it may participate in reinforcing the polarization of blastomeres, and thereby play an important role for compaction and TE lineage formation [3]. These conclusions are based on the findings that depletion of both maternal and zygotic Cdx2 results in developmental arrest at an early blastocyst stage [66]. However, another study reported no correlation between Cdx2 expression and the initiation of TE lineage commitment, suggesting that the contribution(s) of both maternal and zygotic Cdx2 for blastocyst formation still warrants further investigation [4]. The expression of maternal Cdx2 could exist independently of Tead4 and therefore does not rule out the possibility of Tead4 acting upstream of zygotic Cdx2. The development of the ICM is unaffected in *Tead4* mutant embryos, since expression of ICM-specific *trans*-factors including Oct4 and Nanog was detected in *Tead4* mutant embryos, and ES cells could be established from them [65]. However, Tead4 is crucial for the initiation of TE formation since the expression of Oct4 up to the blastocyst stage could be suppressing *Cdx2* expression and thus TE initiation, until Tead4 promotes *Cdx2* expression by overcoming this negative input [64,65]. Moreover, Gata3 expression becomes restricted to TE cells, and this expression is also dependent on Tead4, suggesting that Gata3 and Cdx2 act in parallel pathways downstream of Tead4 and function to activate target genes within the TE lineage [62]. Taken together, these findings reveal a critical requirement for Tead4 in blastocyst formation where a functional TE is one of the hallmarks, and Tead4 is a key transcription factor required for specific activation of *Cdx2* expression and the initiation of TE formation. Although Tead4 regulates *Cdx2*, its expression is not restricted to the outer cells of the developing blastocyst. Rather, Tead4 is ubiquitously expressed in the preimplantation embryo. Hence, the question arises as to whether Tead4 plays an instructive role in TE lineage specification [64].

## 8. Building the TE Lineage through the HIPPO Pathway Signal Cascade

One plausible explanation for how Tead4 might activate *Cdx2* only in outer cells of the compacted morula without being restricted to these cells could be the involvement of a co-activator protein that itself is expressed and/or functions only in these outside blastomeres. Indeed, Tead family transcription factors require the transcriptional co-activator Yes-associated protein (Yap) to stimulate downstream gene expression [65,67,68]. Thus, Yap is a candidate for regulating *Cdx2* expression together with Tead4 in outer blastomeres. Consistent with this hypothesis Yap expression is detected in the nuclei of all blastomeres at the early 8-cell stage, but later on its expression becomes restricted only to outside cells where it increases up until the 30-cell stage and thereafter remains at a constant level [69]. Concomitantly the expression of Yap in inside cells decreases and the protein is excluded from nuclei. Since *Yap* deficient embryos form a normal TE it has been suggested that Taz (transcriptional co-activator with PDZ-binding motif), another transcription co-activator having approximately 50% sequence identity with Yap, might compensate for the loss of Yap [70,71]. High levels of Taz have been observed in the nuclei of outside blastomeres, whereas low levels were detected in nuclei of inside cells [69]. Even though not all Taz-deficient embryos die before birth, double *Yap* and *Taz* mutant embryos exhibit a more severe phenotype as they fail to establishment inside ICM *vs.* outside TE cells [64,72,73]. Thus, co-activators Yap/Taz function to establish the position-dependent Tead4 protein activity underscoring the importance of posttranslational regulation and protein-protein interactions in early development.

**Figure 7:**
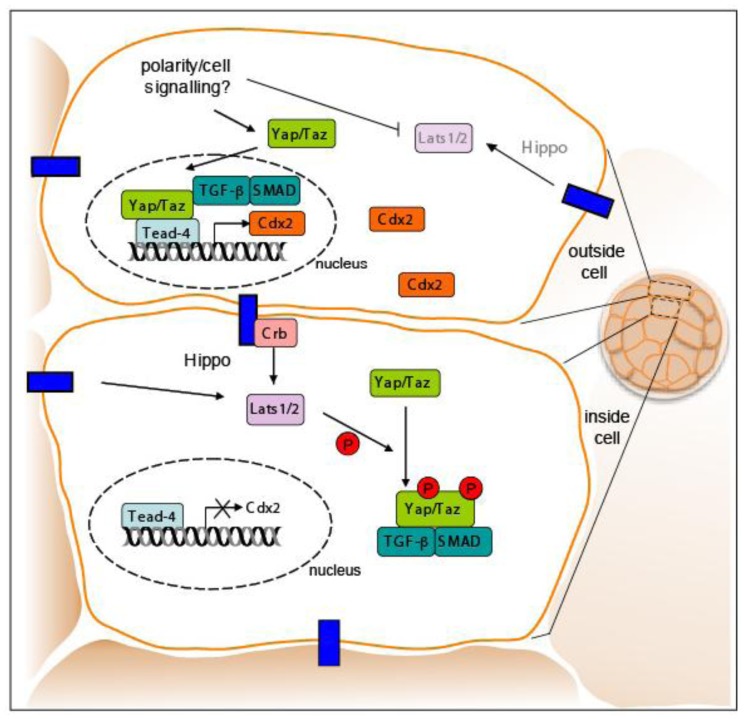
A model of cell position (inside *vs.* outside) and cell fate specification in the early embryo involves members of the Hippo pathway. Several components of the Hippo pathway are implicated in TE *versus* ICM fate. The Hippo pathway converts cell density information into cell growth control and gene activity. In the low-density or “outside” cells, the transcriptional co-factor, Yap/Taz, are trans-located into the nucleus by cellular signals. This nuclear Yap/Taz binds Tead4 and transcriptionally activates TE genes, such as *Cdx2*, to specify TE fate. The Crumbs (Crb) polarity complex interacts with Yap/Taz promoting nuclear accumulation of TGF-B and SMAD signals, linking TFG-B activity in the Hippo pathway. In high-density or “inside cells”, Yap is phosphorylated by Hippo kinases, such as Lats 1/2, excluding Yap/Taz from the nucleus. TGF-B/SMADs are also retained in the cytoplasm. As a consequence, Tead4 remains inactive, keeping TE-specific genes silent. These inside cells are destined to become ICM.

The signaling pathway through which the transcription regulators Yap/Taz have been suggested to establish Tead4 activity is the Hippo pathway (Figure 7). This signaling pathway, which is conserved from *Drosophila* to mammals, is a major regulator of cell growth, proliferation, apoptosis, and is critical for cell fate decisions. Phosphorylation of Yap/Taz by the Ser/Thr kinases Lats1 and Lats2 regulates subcellular localization of the Yap/Taz proteins and thereby their activation of downstream targets [68,69,74]. Phosphorylation of Yap/Taz by Lats1/2 results in accumulation of Yap/Taz proteins in the cytoplasm leading to inactive *Tead4* in inner cells which are fated to form ICM cells. However, in outside cells activated Tead4 induces Cdx2 expression due to shuttling of Yap/Taz into the nuclei of these cells leading to acquisition of TE fate.

Recently a likely connection between the Hippo signaling pathway and the TGFβ/Smad signaling pathway was uncovered when Taz was shown to bind Smad2/3-4 complexes in response to TGFβ-signaling and thus control their nucleo-cytoplasmic shuttling. Active Smad complexes and Yap/Taz proteins co-localized *in vivo* with cell density signals responsible for the phosphorylation of Yap/Taz through binding of the Crumbs polarity complex. Phosphorylation of Yap/Taz leads to the accumulation of cytoplasmic Yap/Taz and suppression of TGFβ signaling [75,76]. The coupling between cell density and Yap/Taz regulation may explain the outside cell restriction of Cdx2. Cdx2 might not be expressed in ICM cells because inside cells, being at a higher density, may sense surrounding cells resulting in phosphorylation of Yap/Taz. The phosphorylation of Yap/Taz in inner cells may fail to activate downstream targets, such as the *Cdx2* gene. Thus, the complex orchestration of *trans*-factors, cell position, cell polarity, and signaling may control TE fate within the early embryo.

## 9. Sorting the Lineages: A Biological Puzzle

Cells of the ICM will commit to either an EPI or PrE fate (Figure 3). It was originally proposed that cells of the ICM were equipotential and could contribute to either EPI or PrE and that fate was primarily controlled by a cell's location within the ICM. This “positional” model primarily arose from the observation that PrE cells form a epithelial monolayer on the surface of the ICM adjacent to the blastocoel cavity [77,78], and was further supported by *ex vivo* studies in which the outer layer of embryoid bodies (generated from ES cells) is composed of extraembryonic endoderm [79,80]. Recently this model has been revised, and it is presently believed that commitment to PrE *vs.* EPI fate occurs at an earlier stage and is likely governed by *trans*-factor expression levels in individual cells, as well as positional signals, cell sorting and selective apoptosis within the ICM [81–83].

## 10. EPI and PrE Lineage Allocation in the ICM

Lineage-specific transcription factors are co-expressed in early ICM cells with both Nanog and Gata6 proteins present in all cells of the embryo until the early blastocyst stage when they are downregulated in outer cells prior to TE formation. However, observations in ES cells have revealed that neither the levels of Nanog nor Gata4/6 are uniform, but in fact they fluctuate over time, with low levels of Nanog predisposing cells towards differentiation even prior to a final commitment [84]. Based on these observations, it is tempting to speculate that this may also be true *in vivo*, though further investigation of *trans*-factor expression levels in individual cells within developing embryos will be required to formally establish if this is the case. Several studies have proposed that commitment to *EPI vs.* PrE fate is evident at, or shortly after, the 64-cell stage. Coincident with this lineage segregation event, Nanog is repressed by Grb2/MAP kinase signaling in a subset of cells, as they initiate PrE lineage commitment [47,81]. A characteristic sequence of *trans*-factor activation is associated with the progression of cells through PrE cell lineage commitment, with a defined temporal and spatial profile: Gata6 > Sox17 > Gata4 > Sox7 [85] (Figure 3). Even though initially widely expressed, Gata6 expression gradually becomes restricted to cells fated to form PrE. From around the ∼64-cell stage the expression of Nanog and Gata6 becomes mutually exclusive resulting in a salt-and-pepper distribution of cells expressing one or other *trans*-factor within the ICM [81,83]. Gata6 expressing cells will co-express Sox17, a transcription factor required for the maintenance of the PrE [85,86]. Indeed, overexpression of Sox17 in individual blastomeres has been shown increase their propensity to give rise to PrE [86,87], whereas overexpression of Gata6 does not [82,86]). Finally, Sox17 has been shown to be required for the isolation of PrE-derived XEN cells highlighting its role on PrE induction [87]. Gata4 is only detected in PrE committed cells once the salt-and-pepper distribution has emerged [81,83], while Sox7 is only detected in PrE-committed cells in their final position on the cavity roof [85]. These findings suggest that within the ICM, EPI *vs.* PrE cell lineage choice occurs at an early time point [81–83]. However, cell fate commitment does not depend on gene expression alone, but is also likely to be influenced and/or reinforced by position within the ICM. Thus perhaps some cell fate plasticity likely exists even after the 64-cell stage.

Prospective Nanog-positive EPI cells are intermingled with prospective PrE cells, which are Gata6/Sox17/Gata4-positive. These two mutually exclusive populations contain cells that are localized in both deeper and more superficial cell compartments of the ICM [81,83]. The salt-and-pepper distribution of prospective EPI and PrE cells is subsequently resolved into two distinct layers as cells of the ICM sort, in a process shown to require differential cell adhesive properties, as well as active actin-dependent movements [82,83]. As sorting proceeds, and PrE fated cells arrive on the cavity roof, these Gata6/Sox17/Gata4-expressing cells become Sox7-positive. At this time any Gata6/Sox17/Gata4-expressing cells that have not sorted to the surface of the ICM, are removed from the embryo through selective apoptosis [82,83]. Gata6/Sox17/Gata4/Sox7-positive cells are positioned on the surface of the ICM adjacent to the blastocyst cavity. From there they concomitantly deposit basement membrane, polarize and initiate epithelialization, until a monolayer of cells is formed on the surface of ICM creating the PrE [83,85]. Thus PrE cells also express polarity and basement membrane markers such as Lrp2, Dab2 and Collagen-IV [88], while Pdgfrα, another early PrE marker, was recently shown to be involved in PrE lineage expansion [89].

## 11. Emergence of a Mutual Distribution of Transcription Factors within the ICM: Still Pieces of the Puzzle Missing

Taken together, the choreography of gene regulation, gene expression, and signaling cascades likely determines cell fate specificity in the early embryo. However, *how* the salt-and-pepper distribution emerges remains unknown, and is subject to much debate. Recently several studies, which are not necessarily mutually exclusive, have been proposed to explain how this distribution might be achieved.

The “time outside - time inside” model proposes developmental timing of cell internalization is key in cell fate choice [86]. Cells from the *first wave* of internalization (from 8-cell to 16-cell stage) have a propensity towards pluripotency and therefore biased towards an EPI fate, whereas cells internalized in the *second* (from 16-cell to 32-cell stage) and possibly also a *third waves* (from 32-cell to 64-cell stage) will be biased towards PrE due to their prolonged external position. This model proposes that pluripotential inversely correlates with “time on the outside”, such that pluripotency is favored when cells are internalized and thus protected from outside (potentially differentiating) factors [86,87,90]. Molecular evidence supporting this model comes from expression levels of both Sox17 and Gata6, which have been reported to be up-regulated in cells internalized from the *second but not the first wave* [86]. Such a situation may indeed be sufficient to prime cells to differentiate to PrE lineage [84,85] This model also posits that cell lineage specification depends on asymmetric cell division arising from cell polarity generated by the different waves of cell internalization. Moreover in a recent study, lineage tracing of embryos using two-photon microscopy revealed that asymmetric cell divisions may arise from a unique cell population, referred to as intermediate, possessing characteristics of both inner and outer cells [91].

Another recent study in which single cells from embryos were expression profiled has suggested that differences in the timing of inner cell formation might give rise to temporal differences in the upregulation of Sox2 within prospective inner cells [40]. In this way, inner cells that are formed earliest activate Sox2 expression before those arising later, and thus these cells have a greater propensity to contribute to EPI. Notably, single cell expression profiling revealed *Fgf4/Fgfr2* expression to be reciprocal before that of lineage-specific *trans*-factors [40]. Thus, both temporal differences in the generation of ICM cells, as well as their Fgf signaling status, may help influence the decision of a cell to adopt an EPI *vs.* PrE fate. In this way, inner cells that form early may preferentially give rise to the EPI lineage, due to high levels of Sox2, which result in the expression of *Fgf4*, a direct target of Sox2 and Oct4 [45,92]. On the other hand, inner cells formed later on would express lower levels of Sox2, and thereby lag in the activation of *Fgf4*, but be exposed to Fgf4 produced by their earlier born counterparts, thereby leading them to adopt a PrE fate. If this is the case, it would argue for a negative feedback mechanism operating within the ICM between cells born from early *vs.* later waves of internalization, such that those already committed to an EPI fate can influence or restrict the developmental fate of the cells that follow them. These findings lend support to the “time outside-time inside” model proposed by Zernicka-Goetz and colleagues [86].

However, yet another recent study has suggested that the timing of generating inner cells may not be sufficient to restrict cell fate commitment [93,94]. Instead a dynamic plasticity may exist within the ICM with the Fgf/MAP kinase-signaling pathway, playing a key role in EPI *vs.* PrE lineage choice. Blocking Fgf/MAP kinase signaling, either in *Grb2*-deficient embryos or by addition of Fgfr/MEK inhibitors, resulted in all ICM cells adopting an EPI identity. By contrast, all ICM cells acquired a PrE identity when embryos were treated with a high dose of exogenous Fgf The importance of Fgf/MAP kinase signaling in the segregation of EPI and PrE lineages is further supported by earlier studies on embryos deficient in various pathway components including the ligand Fgf4, the Fgf receptor 2 (*Fgfr2*) a receptor tyrosine kinase (RTK) and *Grb2* (an RTK adaptor protein) [81,95–98]. Although there is evidence for this biased development, it does not mean that cells are fully committed and not flexible to respond to extreme signaling conditions, such as high Fgf activity or loss of Fgf signaling [93,94]. Even though these two models may seem contradictory regarding how the proportion of EPI *vs.* PrE lineages is regulated in the early preimplantation mouse embryo, this disparity has been discussed [99–101]. Indeed, technical differences could lead to apparent discrepancies between studies. Yamanaka and colleagues were analyzing the contribution of fluorescent labeled progeny of single 8-cell blastomeres in both pre- and post-implantation embryos, the latter ensured that the EPI/PrE cells were fully functional and did commit to the different cell fates. By contrast, Morris and colleagues were manually tracking each cell in 8-cell blastomeres. This allowed them to detect which of the cells had originated from the first *vs.* second wave of internalization and thereby analyze the proportion of EPI/PrE lineages based on cell position. The key differences between the two studies may lie in the proportions of internalized cells in each wave of asymmetric division. Based on these findings the different outcome could either be due to different mouse strains used in these two studies, where CD1 may divide more asymmetrically than C57Bl/6;CBA hybrids, or simply arise from the different experimental approaches [99–101].

## 12. Inducing the Early Lineages by Reprogramming Adult Somatic Cells: The Importance of Transcription Factors

The first ES cells were isolated over thirty years ago by Evans and Kaufman and Martin [20,21]. Their experiments described the methods to derive and maintain ES cells indefinitely in a pluripotent state as defined by the formation of a germline transmitting chimeras. These groundbreaking studies with ES cells led to the formulation of methods for gene targeting and the generation of genetically modified strains of mice [1,2]. We now know that the *trans*-factors specific for each lineage of the blastocyst stage embryo, play important roles in the establishment and maintenance of stem cell types representing each cell lineage of the blastocyst. Loss of Oct4 in ES cells results in a loss of pluripotency and cell fate, underscoring the importance for this factor in “stem-ness”, therefore, Oct4 levels must be tightly regulated [41,102]. Similarly, overexpression of Cdx2 or Gata3 in ES cells can completely override the pluripotency program and direct cells into a TS cell state [54,62], while XEN-like cells arise from overexpression of Gata4 or Gata6 in ES cells [53,103]. Indeed it has also been shown that a crucial ES cell maintenance factor, Myc, can repress PrE differentiation through Gata6 [104]. Taken together, lineage-specific *trans*-factors together with co-factors target genes to turn on a program defining cell lineage identities.

Stem cell biology has rapidly advanced in recent years due to the characterization of transcription factors that serve as key regulators for different lineages. In their landmark study, Yamanaka and Takahashi showed that overexpression of Oct4, Sox2, Nanog, Klf4, and c-Myc is sufficient to reprogram adult somatic cells back to an induced pluripotent stem cell (iPS) state [1,2,105]. These studies paved the way for experiments in human cells, as well as for lineage-specific cellular reprogramming, and now offer hope for correcting debilitating diseases [106].

## 13. Setting the Marks in the Embryo

It is well established that ectopic expression of single or multiple *trans*-factors can be sufficient to convert one cell type into another, as was originally demonstrated with the studies of Weintraub and colleagues nearly twenty five years ago when they converted fibroblasts to myoblasts by ectopic expression of MyoD [107]. The embryonic and extra-embryonic lineages are established by key *trans*-factors such as Oct4, Sox2, Nanog, Cdx2, and Eomes. Once specified, these cell fates must be stably inherited. The exact molecular mechanisms driving lineage allocation in the developing embryo and iPS reprogramming events remain largely unknown. It is likely that epigenetic modifications and an epigenetic code in addition to the *trans*-factors are reset to alter cellular fate.

The prefix “Epi” (Greek for “on top of” or “in addition to”) in epigenetics literally refers to a mechanism that leads to a stable, yet reversible, phenotype without a change in genotype [38]. The term epigenetics has several definitions and at times the use of the term “epigenetics” may be misleading or misused as essayed by Ptashne and Bird [108,109]. In 1957, Conrad Waddington defined epigenesis as the study of how cells give rise to phenotypes during development [110]. Waddington's view of epigenetics described the development of a cell lineage from a pluripotent state towards terminal differentiation as the path of a ball travelling down several branching pathways, where the ball once reaching the final valley cannot return back up to hill to its starting point. The generation of iPS cells, nuclear transfer and somatic cell fusions defy Waddington's original definition of epigenetics by reprogramming a terminally differentiated cell back to an ES-like state. More recently Riggs and colleagues defined epigenetics as, “the study of mitotically and/or meiotically heritable change in gene function that cannot be explained by changes in DNA sequence” [111]. Taken together, epigenetics is a complex regulatory mechanism irrespective of the nucleic acid code that is not well understood in mammals. Here, we discuss the stably, inherited epigenetic marks including DNA methylation and chromatin modifications in the early mouse embryo. DNA methylation and polycomb group proteins (PcG) are two classic epigenetic systems in development [108].

In mammalian development, X-chromosome inactivation (XCI) and genomic imprinting are examples of epigenetic regulation. XCI, a crucial epigenetic event in early mammalian development, is accomplished by randomly silencing one of the two female X chromosomes in the soma to ensure equal X gene expression between XX females and XY males. A second form of XCI occurs in the extraembryonic tissues (imprinted XCI), in which the paternal female X chromosome is silenced [112,113]. These two forms of XCI segregate within cell lineages. Shortly after fertilization, the paternal X-chromosome is inactivated. This inactive state must be erased within cells of the embryo proper before random XCI takes place. Between E3.5 an E4.5, the inactive paternal X chromosome is exclusively reactivated in cells fated to form the pluripotent epiblast, but not in extraembryonic cells, namely those fated to form trophectodem (TE) and primitive endoderm (PrE). Shortly after this time, one of the two Xs is randomly chosen for silencing. Each of the stem cell types derived from, and representing, the lineages of the blastocyst faithfully recapitulates the events taking place within the embryo (ES cells exhibit random, whereas XEN and TS cells show the imprinted form of XCI). XCI exemplifies the global epigenetic reprogramming observed during ES cell differentiation. Somatic XCI is achieved by homologous X-chromosome pairing, counting, and the mutually-exclusive choice of active *vs* inactive X chromosome. XCI is tightly coupled with ES differentiation where Oct4 regulates XCI by triggering X-X pairing and counting [114]. These results agree with data proposing that both Oct4 and Nanog regulate XCI [115]. The epigenetic reprogramming of somatic cells to iPS is accompanied by the reactivation of the silenced female X chromosome [116]. Taken together, these observations couple ES cellular differentiation with XCI via the pluripotent factors, such as Nanog and Oct4.

Genomic imprinting is an epigenetic process set in the germ line that allows parent-of-origin gene expression. One of the most studied imprinted loci is the mouse *H19/Igf2* domain on chromosome 7. *H19* and *Igf2* show monoallelic expression from the maternal and paternal chromosomes, respectively [117]. Both XCI and autosomal imprinting share common molecular mechanisms such as DNA methylation, long non-coding RNAs, DNA repeat regions, and chromatin insulation [118]. DNA methylation is an essential process in mammalian development and is achieved by the placement of 5-methylcytosine (5mC) on CpGs via DNA methyltransferase enzymes [119–121] Approximately ∼2–8% of the total cytosine's in the mammalian genome are methylated resulting in a broad range of biological function such as chromatin structure, gene expression, and the maintenance of cellular identity. DNA methylation is associated with stable gene silencing such as the marks present on the inactive X female chromosome [113].

Three catalytically active DNA methyltransferases (Dnmt)1, Dnmt3a, and Dnmt3b establish and maintain DNA methylation in mammals. The *de novo* DNA methyltransferases Dnmt3a and Dnmt3b initially establish DNA methylation during the blastocyst stage of development while the maintenance methyltransferase Dnmt1 maintains DNA methyl marks during cell divisions. In addition, two homologous proteins, Dnmt2 and Dnmt3L are expressed in several cells types including ES cells [111]. Loss of *Dnmt1* and *Dnmt3b* in mice results in embryonic lethality at E8.5-9 [122,123]. whereas *Dnmt3a* null mice die by four weeks of age [123]. Taken together, the DNA methyltransferases are crucial for early mouse development. Although DNA methylation is generally regarded as a stable epigenetic mark, DNA demethylation may remove the 5-methylcytosine. In early development fertilization initiates epigenetic events that are characterized by rapid active DNA demethylation prior to DNA replication and by the time of the first lineage segregation and differentiation of TE, *de novo* DNA methylation is initiated again in the embryo. Indeed, one of the earliest epigenetic marks in lineage allocation is this global wave of *de novo* methylation of the ICM in blastocysts. In contrast, the TE cells in the blastocyst remain hypomethylated [124]. This differential methylation is retained through development in these tissues [125,126]. The Hemberger and Reik lab's reported that embryonic stem cells deficient for the maintenance DNA methyltransferase, *Dnmt1*, results in trophoblast differentiation [127]. They further showed that the *Elf5* transcription factor promoter is differentially methylated: hypermethylated in wild type ES cells and hypomethylated in wild type TS cells. The loss of *Dnmt1* in ES cells, results in hypomethylation of the *Elf5* promoter, suggesting that Elf5 may directly activate the TS genes. Consistent with this hypothesis, Elf5 can bind and reinforce expression of the TS genes *Cdx2* and *Eomes* [127]. Thus, DNA methylation is an important TE *versus* ICM cell fate switch.

Other classical epigenetic systems in development are the polycomb group (PcG) and trithorax group (TrxG) proteins. These names derive from the mutant phenotypes in the fruitfly and these modifiers maintain active gene repression (PcG) or activation (TrxG) states [128,129]. In addition to all of the DNA methyltransferases, the PcG proteins are highly expressed in undifferentiated mouse ES cells [130]. Whereas the loss of DNA methylation prevents ES cells from stably differentiating, the loss of PcG proteins results in differentiation. As mentioned above, one of the earliest lineage segregation marks are histone modifications [35]. In the mouse, the PcG proteins catalyze the H3K27me3 histone modification. This is accomplished by polycomb repressive complex (PRC) 2 that contains enhancer of zeste (Ezh2) (the enzyme that tri-methylates H3K27), Eed (embryonic ectoderm development), and Suz12 (suppressor of zeste 12). The loss of any of the PRC2 subunits in the mouse results in early embryonic lethality underscoring their importance. *Ezh2*-deficient embryos die around gastrulation [131,132]. *Eed*-deficient embryos have gastrulation defects and do not maintain the imprinted form of XCI in the extraembryonic tissues [133,134]. ES cells lacking *Eed* show spontaneous differentiation and exhibit derepressed developmental genes [135–137]. *Suz12* mutants show early postimplantation defects [131,132].

The recent demonstration that the ten-eleven translocation (Tet) proteins can hydroxylate 5mC to 5-hydroxymethylcytosine (5hmC) raised the possibility that an additional epigenetic state may exist [138]. Mouse ES cells have a relative enrichment of 5hmC and the Tet1 family member is highly expressed in undifferentiated ES. *Tet1* mRNA levels decrease upon ES cell differentiation [139]. Tet1 is required for ES cell maintenance [139] as knockdown of *Tet1* in ES cells results in the upregulation of *Cdx2, Sox17*, and other genes involved in development and differentiation. In contrast, ablation of *Tet1* in ES cells results in a decrease of genes related to pluripotency and ES cell function [140]. Knockdown of *Tet1* in pre-implantation embryos results in a bias towards TE, suggesting that Tet1 is important for ICM specification [139]. Tet1 preferentially binds the transcriptional start sites at CpG-rich promoters at both active and polycomb repressed genes, establishing a role of Tet1 in modulating DNA methylation [141].

Taken together, DNA methylation and histone modifications are essential for early mouse lineage specification. Epigenetic landscapes set the stage for understanding normal development, as well as understanding the reprogramming of somatic cells to pluripotent states.

## 14. What More Can the Mouse Teach Us?

We have made great strides in interrogating the early mouse embryo towards better understanding how the respective lineages are derived and maintained. Nevertheless, many questions still remain. To date, there are several key lineage-specific transcription factors shown to be involved in the allocation and/or maintenance of the first cell lineages (TE, EPI, and PrE). However, neither all their downstream gene targets nor their protein interacting partners have been identified. Also, we now recognize that the levels of these *trans*-factors are important, but we do not yet fully understand how the fluctuations of some of these proteins are regulated, or what they signify. Might post-translational modifications of these *trans*-factors alter their activity? We still need to develop a detailed understanding of the interactions taking place between different factors operating within the gene regulatory networks contributing to the complex signaling pathways responsible for lineage specification. Moreover, we would need better insight into how transcriptional regulation is coupled to the control of cell position, cell density and cell signaling. Furthermore, epigenetic events can contribute to cell fate decisions in the early embryo, but it is still unknown how these lineage-specific *trans*-factors remodel the chromatin in the context of epigenetic changes or remodeling within specific genic locations to induce a particular cell fate.

Studies in both mouse and human have identified *trans*-factors necessary and sufficient to convert a differentiated somatic cell back to the pluripotent state (induced pluripotent stem cell; iPS cell). But this is an inefficient process, taking weeks for an adult cell to revert back to as state of “stem-ness”. Indeed, additional investigations are needed to understand this conversion process to drive cells to a particular cell fate for replacement therapies. A primary goal of iPS cell methodology is to study diseases in a cell culture system as well as to deliver and repopulate damaged tissues (such as those in Parkinson's disease, amyotrophic lateral sclerosis, *etc.*) in order to restore normal function [142]. The exciting possibility of taking patient autologous cells, correcting an inherent genetic deficiency in an *ex vivo* system, and subsequently transplanting these corrected cells back to the patient without tumor formation is currently not possible but a hopefully an attainable goal for the future. Thus, both the efficacy and the delivery of properly fated cells are crucial for the advancement of this field to human therapies. The crucial marks that get reset upon conversion to a state of “stem-ness” are presently under investigation and the molecular mechanisms that “erase” these adult lineage marks to the progenitor state are not fully understood.

To understand how pluripotency is regulated, we need to understand the mechanisms deployed by the embryo to control lineage choice and plasticity. Not only on the molecular level, but also epigenetically since the early mouse embryo and its pluripotent cells have a characteristic epigenetic signature to mark cell fate that is subject to reprogramming. Thus, the mouse provides us with a model to study the acquisition of an iPS cell-like state and therefore offers a powerful tool for regenerative medicine [38]. Taken together, understanding the mouse embryo should lead to a deeper understanding of human stem cells, which will provide new insights in how these can be exploited for treatment and therapy. We still have much to learn from the mouse.
